# The Role of Egg Production in the Etiology of Keel Bone Damage in Laying Hens

**DOI:** 10.3389/fvets.2020.00081

**Published:** 2020-02-21

**Authors:** Beryl Katharina Eusemann, Antonia Patt, Lars Schrader, Steffen Weigend, Christa Thöne-Reineke, Stefanie Petow

**Affiliations:** ^1^Institute of Animal Welfare and Animal Husbandry, Friedrich-Loeffler-Institut, Celle, Germany; ^2^Institute of Farm Animal Genetics, Friedrich-Loeffler-Institut, Mariensee, Germany; ^3^Institute of Animal Welfare, Animal Behavior and Laboratory Animal Science, Freie Universität Berlin, Berlin, Germany

**Keywords:** hen, keel bone, fracture, deviation, radiographic density, laying performance, egg production

## Abstract

Keel bone fractures and deviations belong to the most severe animal welfare problems in laying hens and are influenced by several factors such as husbandry system and genetic background. It is likely that egg production also influences keel bone health due to the high demand of calcium for the eggshell, which is, in part, taken from the skeleton. The high estrogen plasma concentration, which is linked to the high laying performance, may also affect the keel bone as sexual steroids have been shown to influence bone health. The aim of this study was to investigate the relationship between egg production, genetically determined high laying performance, estradiol-17ß concentration, and keel bone characteristics. Two hundred hens of two layer lines differing in laying performance (WLA: high performing; G11: low performing) were divided into four treatment groups: Group S received an implant containing a GnRH agonist that suppressed egg production, group E received an implant containing the sexual steroid estradiol-17ß, group SE received both implants, and group C were kept as control hens. Between the 12th and the 62nd weeks of age, the keel bone of all hens was radiographed and estradiol-17ß plasma concentration was assessed at regular intervals. Non-egg laying hens showed a lower risk of keel bone fracture and a higher radiographic density compared to egg laying hens. Exogenous estradiol-17ß was associated with a moderately higher risk of fracture within egg laying but with a lower risk of fracture and a higher radiographic density within non-egg laying hens. The high performing layer line WLA showed a significantly higher fracture risk but also a higher radiographic density compared to the low performing layer line G11. In contrast, neither the risk nor the severity of deviations were unambiguously influenced by egg production or layer line. We assume that within a layer line, there is a strong association between egg production and keel bone fractures, and, possibly, bone mineral density, but not between egg production and deviations. Moreover, our results confirm that genetic background influences fracture prevalence and indicate that the selection for high laying performance may negatively influence keel bone health.

## Introduction

Keel bone damage (KBD) is one of the most serious animal welfare problems in laying hens (1–3). The term comprises fractures and deviations of the keel bone, i.e., the ventral part of the sternum in birds. Keel bone fractures can affect up to 97% of hens within one flock (4–8), and it is likely that these fractures are painful (9, 10). The prevalence of deviated keel bones, being defined as “bone[s] with an abnormally shaped structure that has not resulted from a fracture but contains section(s) that vary from a theoretically perfect 2-dimensional straight plane in either the transverse or sagittal planes. Additionally, indentations along the ventral surface can also be classified as a deviation” (11), can reach up to 82% (12, 13). Age (4, 5, 14–18), different housing systems (4, 7, 19), nutrition (20–22), and genetic background (5, 17–19, 23, 24) have been shown to influence KBD. Egg production and the high laying performance of modern laying strains may also favor the occurrence of keel bone damage. There is a high calcium demand for the eggshell. The skeleton is an important source of calcium, especially the medullary bone, a special kind of woven bone which is found in the medullary cavity of female birds and which serves as a labile source of calcium (25, 26). It is suggested that the osteoblasts switch from building structural, i.e., cortical and trabecular bone, to building medullary bone when hens reach sexual maturity and that, thus, no formation of structural bone occurs during lay while resorption of structural bone continues, leading to a progressive bone loss (27). However, no detailed studies about the role of egg production in keel bone damage are available so far. In a previous work that aimed at testing the influence of the GnRH agonist deslorelin acetate on reproductive physiology in laying hens, we found a lower prevalence of keel bone fractures and a smaller proportion of deviated keel bone area in hens that were prevented from egg laying by a sustained release deslorelin acetate implant compared to control hens (28). However, the effect of treatment with deslorelin acetate on keel bone health could also have been mediated via other pathways that are not dependent on egg production. For example, treated hens in the previous study showed a decreased estradiol-17ß plasma concentration compared to control hens. This hormone plays an important role in bone structure, bone metabolism, and bone diseases in chickens (29, 30). It is assumed that it is the rise in estrogen plasma concentration at the onset of lay that stimulates the osteoblasts to form medullary rather than structural bone, leading to depression in structural bone formation and to osteoporosis (31). Furthermore, weaker bones with a thinner cortex and large defects within the cortical bone were found in hens treated with exogenous estradiol compared to untreated hens (32). Reduced bone strength and cavity formation in the cortical bone were also found after treatment with exogenous estradiol in roosters (32) and capons (30). Thus, differences between treated and control hens in our previous study may have additionally been caused by different estradiol concentrations and possibly other factors that were influenced by deslorelin acetate, and not by egg production alone.

There are also only few studies on the high laying performance that modern laying strains have been selected for and its possible role in KBD. Hocking et al. (33) compared commercial breeds with a high laying performance and traditional breeds with a significantly lower laying performance and found a higher radiographic density of keel bones and tibiotarsi and also a higher breaking strength of humeri and tibiotarsi in traditional compared to commercial breeds. Furthermore, Candelotto et al. (24) compared the risk of experimental keel bone fractures in an experimental line that descended from a dam line which had not been selected for any breeding goal for several years and a sire line which had been bred for dual egg and meat production to the risk in layer lines that had been selected for high productivity. They found a lower number of experimental fractures in the experimental line (24). Similarly, we found a higher prevalence of keel bone fractures and more severe keel bone deviations in a high compared to a low performing brown layer line in a previous study (19). However, in the same study, no such clear differences between high and low performing layer lines were found within the white layers (19).

When investigating keel bone damage in laying hens, it is crucial to observe the same hens over a longer period of time because prevalence of both keel bone fractures and deviations has been shown to increase with age (5, 14, 16–19). However, in some studies, prevalence only increased until about the 50th week of age and then leveled off or even decreased (4, 15, 21). Although it is possible that this peak in prevalence of fractures and deviations may partly be explained by the decreasing pool of hens that are still fracture and deviation free, it is also possible that the keel bone is less susceptible to damage after a certain age. Thus, the exact influence of age on keel bone damage and the mechanism behind it still remain to be examined.

The aim of the current study was to experimentally investigate the potential influence of egg production, selection for high laying performance, estradiol-17ß, and age on keel bone fractures and deviations as well as radiographic density of the keel bone.

We additionally addressed the locomotor activity of the hens because this may influence the prevalence of KBD as well. Physical activity has been shown to lead to a higher radiographic density, a higher amount of cortical and cancellous bone, and a higher breaking strength of different bones (16, 34–36). However, keel bone fracture prevalence was found to be higher in more active housing systems such as floor housing and aviaries compared to cage systems (4, 7, 19, 37). This phenomenon is usually explained by the higher risk of collisions with housing equipment that may lead to fractures (7, 38). Thus, we aimed at investigating whether egg laying and non-egg laying hens as well as high and low performing layer lines differed in their level of locomotor activity in order to account for this potential confounding factor in prevalence of KBD. Locomotor activity could differ between egg laying and non-egg laying hens in two opposite directions: On the one hand, egg-laying hens could show decreased locomotor activity levels compared to non-egg laying hens in order to compensate for the energy costs of egg production. This has been suggested for zebra finches by Williams and Ternan (39) who found lower locomotor activity levels in breeding compared to non-breeding pairs. On the other hand, administration of gonadal steroids such as testosterone or estradiol has been found to increase locomotor activity in castrated male Japanese quail (40) and in ovariectomized rats (41), indicating that these hormones have a large effect on locomotor activity. Since, based on results of a previous study (28), egg laying hens were supposed to show higher estradiol-17ß plasma concentrations compared to non-egg laying hens, they could also show higher, estrogen-mediated locomotor activity levels. Furthermore, we suspected egg laying hens but not non-egg laying hens to show nesting behavior which has been shown to be mediated by estrogens and progesterone (42). As nesting behavior includes increased activity levels prior to oviposition (43, 44), this could also result in an increase in general activity throughout the day in egg laying compared to non-egg laying hens. Thus, we hypothesized that general locomotor activity would differ between treatment groups but did not speculate about the direction of this difference as both possibilities, i.e., decreased or increased locomotor activity levels in egg laying compared to non-egg laying hens, are plausible.

Taken together, we hypothesized that

1. non-egg laying hens would show a lower risk of keel bone fracture and deviation, less severe keel bone deviations, and a higher radiographic density of the keel bone compared to egg laying hens.
2. exogenous estradiol-17ß would increase the risk of keel bone fracture and deviation as well as the severity of deviations in egg laying and non-egg laying hens.
3. hens of a low performing layer line would show a lower risk of keel bone fracture and deviation, less severe keel bone deviations, and a higher radiographic density of the keel bone compared to hens of a high performing layer line.
4. the prevalence of keel bone fractures and deviations as well as the severity of keel bone deviations would increase with age.
5. locomotor activity would differ between treatment groups.

## Materials and Methods

### Birds and Housing Conditions

The experiment was performed in accordance with the German Animal Protection Law and approved by the Lower Saxony State Office for Consumer Protection and Food Safety (No. 33.19-42502-04-15/1966).

We examined two different but genetically closely related purebred White Leghorn lines of laying hens (*Gallus gallus domesticus*) which differ in laying performance: Layer line WLA originates from a breeding line of Lohmann Tierzucht GmbH, Cuxhaven, Germany selected for laying performance. The line has been maintained without selection since 2012 at the Friedrich-Loeffler-Institut, Institute of Farm Animal Genetics, Mariensee, Germany. Hens of this line lay around 320 eggs per year. The other line, G11, kept at the institute since 1965 as a conservation flock, is a low performing layer line with an average laying performance of 200 eggs per year.

All chicks (WLA: *n* = 256, G11: *n* = 235) were hatched on the same day and raised in a floor housing system. Birds of the different layer lines were kept in two separate rearing compartments of 23 m^2^ each that were littered with wood-shavings and straw. Perches were provided from the 4th week of age onwards. A standard light program was applied throughout the rearing period and a conventional complete feed for chicks (until 7 weeks of age; 12.97 MJ AMEn/kg DM, 189.61 g/kg crude protein, 31.38 g/kg crude fat, 9.14 g/kg Ca, 6.94 g/kg P) and pullets (from 8 to 19 weeks of age; 12.82 MJ AMEn/kg DM, 151.67 g/kg crude protein, 30.21 g/kg crude fat, 15.83 g/kg Ca, 8.11 g/kg P) as well as water were offered *ad libitum*.

At 11 weeks of age, males were separated from the group and 100 female pullets per layer line were relocated to the experimental site where they were kept for the remainder of the experiment. There were two pens per layer line resulting in 50 hens per pen. All four pens were located in the same poultry house and were set up in the same way: Each pen measured 11 m^2^, was littered with wood-shavings and straw and equipped with perches and a nest box. There were four mushroom-shaped plastic perches per pen. Each perch measured 205 cm × 7 cm × 5 cm (length × height × width at the top, i.e., where the feet are in contact with the perch). All perches were installed at a height of 60 cm and the distance between two perches measured 25 cm while the distance between the wall and the first perch measured 20 cm. There was one wooden nest box per pen which measured 92 cm × 76 cm × 60 cm (length × height × width) and which was installed at a height of 70 cm. For alleviated access to the nest box, two squared wooden perches (92 cm × 3 cm × 5 cm) were installed in front of the nest box and at the same height. The distance between both perches as well as the distance between the first perch and the nest box measured 10 cm. Duration of the light period increased gradually from 10 h/d (until the18th week of age) to 14 h/d (from the 24th week of age onwards). All laying hens were fed *ad libitum* on a conventional laying hen diet (11.68 MJ AMEn/kg DM, 168.11 g/kg crude protein, 29.43 g/kg crude fat, 50.05 g/kg Ca, 5.06 g/kg P) and had *ad libitum* access to water.

### Treatment

There were four different treatment groups per layer line. Thirty-eight hens per layer line (group S) were administered an implant containing 4.7 mg of the gonadotropin-releasing hormone (GnRH) agonist deslorelin acetate (Suprelorin^®^, Virbac, Carros, France). Twelve hens per layer line (group E) were administered an implant containing 75 mg of the gonadal steroid estradiol-17ß (Innovative Research of America, Sarasota, Florida, USA). Twelve hens per layer line were administered both implants (group SE) and thirty-eight hens per layer line were kept as control hens (group C) and did not receive any implant or sham handling. Unbalanced sample sizes were due to the high costs of the estradiol-17ß implant, which only allowed for a comparably low number of hens treated with this implant. Within the two pens per layer line, the four treatment groups were equally allocated, resulting in 19 S, 6 E, 6 SE, and 19 C hens per pen. Both implants, deslorelin acetate and estradiol-17ß, are sustained release implants which continuously emit their active component. The deslorelin acetate 4.7 mg implants have been shown to inhibit follicle maturation and thereby egg production in laying hens for about 12 weeks in a previous study (28). The estradiol-17ß implants are declared by the manufacturer to emit the steroid hormone for 90 days. Based on the results of a previous study (28), hens were first implanted shortly after the onset of lay. As age at onset of lay differed between the layer lines, WLA received the first implant in the 25th week of age while G11 received the first implant in the 27th week of age. Throughout the experimental period, administration was repeated every 90 days (three successive implants in total).

All implants were administered subcutaneously. Hens were anesthetized with 2–3% isoflurane (CP-Pharma Handelsgesellschaft mbH, Burgdorf, Germany) in compressed air with a flow rate of 500 ml/min delivered via face mask. Before application, the application site (first implantation: between vertebral column and left scapula; second implantation: between vertebral column and right scapula; third implantation: left knee fold) was aseptically prepared. In case of administration of a deslorelin acetate implant, the implant was administered with the aid of an applicator that had been delivered together with the implants and the implantation site was sealed with a tissue adhesive (Surgibond^®^, SMI, St. Vith, Belgium). In case of application of an estradiol-17ß or both implants, the skin was cut with surgical scissors at a length of about 2 cm and the implant was administered subcutaneously with forceps. The implantation site was then sealed with two to three simple interrupted stitches.

### Ultrasonography of Ovaries

To verify that hens treated with deslorelin acetate or both implants (deslorelin acetate and estradiol-17ß) did not lay eggs, each hen was examined via ultrasonography every 3 weeks to check for ovarian follicles. The examination was conducted with the ultrasound system DUS 60 vet and the microconvex transducer C611-2 (both Edan Instruments GmbH, Shenzhen, China) as previously described (28).

### Periodical Sampling

During the experimental period of a total of 50 weeks, seven sampling periods of 4 weeks each (sampling period 7 = 3 weeks only) were defined (Table 1). In each of these sampling periods, each hen was radiographed and 2–3 blood samples were collected (see below). Additionally, the activity of the hens was measured during the last 2 weeks of each sampling period.

**Table 1:** Schedule of the experiment with the seven sampling periods, the corresponding weeks of age, and the experimental procedures that were carried out in the respective week of age.

**Week of age**	11	12	13	14	15	16	17	18	19	20	21	22	23	24	25	26	27	28	29	30	31	32	33	34	35	36
Procedure		B	B R	A	A				B	B B R	A	A				B	B B R	A	A			B	B B R	A	A	
Sampling period		1							2							3						4				
Week of age	37	38	39	40	41	42	43	44	45	46	47	48	49	50	51	52	53	54	55	56	57	58	59	60	61	62
Procedure			B	B B R	A	A							B	B B R	A	A								B	B B R A	A
Sampling period			5										6											7		

*B, blood sampling (two B in one cell mean that blood samples were collected twice within the respective week); R, radiography of the keel bone; A, locomotor activity assessment*.

A subgroup of hens (6 S and 6 C hens both of each layer line) was euthanized for another project after sampling period 6. Hence, and due to animal losses as well as parameters that could not be assessed for some hens in some sampling periods (e.g., fractures could not certainly be detected or excluded when the legs were overlapping with the keel bone in the radiograph), the number of hens varied between sampling periods and parameters. The numbers of hens of each layer line and treatment group that were included in the analysis of each parameter within a certain sampling period are given in the results section.

### Weighing of the Hens

Each hen was weighed before each blood withdrawal (= 2 to 3 times per sampling period) and the mean between all values within one sampling period was calculated to get the mean body weight of each hen per sampling period.

### Radiographic Examination of the Keel Bone

All hens were radiographed seven times throughout the experiment (= once per sampling period).

Digital radiographs were taken and evaluated as previously described (19). The non-anesthetized hen was gently placed on its left side on the digital flat panel detector Thales Pixium 2430 EZ wireless (Thales Electron Devices S.A., Vélizy-Villacoublay, France) and lateral radiographs were taken with 50.0 kV and at 2 mAs using the X-ray apparatus WDT Blueline 1040 HF (Wirtschaftsgenossenschaft deutscher Tierärzte eG, Garbsen, Germany) and the X-ray suitcase Leonardo DR mini (Oehm und Rehbein GmbH, Rostock, Germany). To deduce the radiographic density of the keel bone (see below), an aluminum step-wedge was radiographed together with each hen for calibration purposes. One person (SP) blindly evaluated all images for the presence of fractures, using the image processing system AxioVision 4.8 (Carl Zeiss Microscopy GmbH, Jena, Germany). Another person (BE) blindly evaluated all images for the presence and dimension of deviations and for radiographic density, using the image processing program ImageJ (National Institutes of Health, Bethesda, Maryland, USA).

#### Fractures

Each radiograph was evaluated for the presence of one or several keel bone fractures. These were either seen as areas of the bone with callus formation (old fractures) or as black thin lines without callus formation (new fractures). A radiograph was scored 1 if one or multiple fractures were visible (regardless whether it was an old or a new fracture) and 0 if no fractures were visible.

#### Deviations

**Figure 1:**
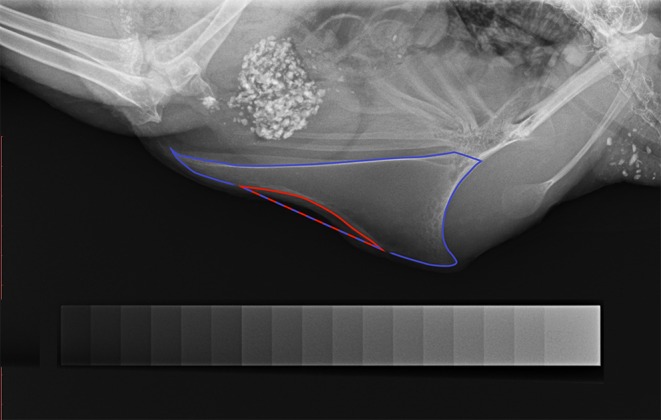
Radiograph of a deviated keel bone. The keel bone surface area is circumscribed with blue color; the area of deviation is circumscribed with red color. The blue-red line marks the straight line between both extremes of the deviated outline. The aluminum step-wedge can be seen at the bottom of the figure.

Each radiograph was evaluated for the presence of a deviation and scored 1 if the keel bone was deviated and 0 if the keel bone was not deviated. Further, the severity of a deviation was estimated by calculating the proportion of the deviated keel bone area (POD). The deviated area was circumscribed along the deformed outline and both extremes of this outline were linked by a straight line as an estimate for the size of the deviated keel bone area (Figure 1). The size of this area was calculated by ImageJ. Afterwards, the whole keel bone was circumscribed up to the insertion of the trabecula intermedia and the size of its surface area was calculated by ImageJ. Again, both extremes of the deformed outline were linked with a straight line as an estimate for the size of the assumed total keel bone surface area (Figure 1). Finally, POD was calculated as the proportion of deviated keel bone area in relation to the assumed total keel bone surface area in percent.

#### Radiographic Density

The method to assess radiographic density of the keel bone was similar to a method described by Fleming et al. (45) who assessed radiographic density of the humerus in laying hens. In the present study, an aluminum step-wedge was radiographed together with each hen for calibration purposes (Figure 1). The step-wedge consisted of 17 steps with thickness ranging from 0.5 to 4.5 mm (in 0.25 mm-increments). The gray value of the background and of all 17 steps was measured after which a calibration curve was generated with a 3rd degree polynomial function. Based on this calibration curve, keel bone radiographic density was assessed, circumscribing the whole keel bone up to the insertion of the trabecula intermedia. The mean gray value was given as millimeters of aluminum equivalent (mm Al eq). Callus formation and legs overlapping with parts of the keel bone resulted in increased, non-representative density measures. Thus, such areas were excluded from radiographic density assessment.

### Measurement of Estradiol-17ß Concentration in Plasma

Blood samples of all 200 hens were collected on 3 days within one sampling period. In sampling period 1, blood samples were only taken twice because the animals were young and blood volume was still low. All blood samplings took place between 8 a.m. and 1 p.m. A maximum of 2 ml blood was taken from the ulnar vein and collected in a test tube coated with Ethylenediaminetetraacetic acid (EDTA) as an anticoagulant (VACUETTE^®^ EDTA tubes, Greiner Bio-One GmbH, Frickenhausen, Germany). Immediately after sampling, blood samples were centrifuged at 3,500 rpm at 4°C for 10 min (Centrifuge Z 300 K, HERMLE Labortechnik GmbH, Wehingen, Germany) and stored at −20°C until further analysis.

Estradiol-17ß concentration was measured in pg/ml using a commercial enzyme-linked immunosorbent assay (ELISA) kit (IBL International GmbH, Hamburg, Germany). The analysis was performed following the instructions of the kit. A pool plasma sample was included on each kit together with the individual samples to calculate the inter-assay coefficient of variation, which was 6.72%. Each blood sample was measured in duplicate. Thereby, the intra-assay coefficient of variation was calculated (mean of all intra-assay coefficients of variation: 1.96%). The mean of both values of the duplicate was defined as the estradiol-17ß concentration of the hen for the specific day. The mean of the two (sampling period 1) or three (sampling periods 2–7) day concentrations within one sampling period was then calculated and defined as the estradiol-17ß plasma concentration of the hen within the given sampling period.

### Locomotor Activity Assessment

**Figure 2:**
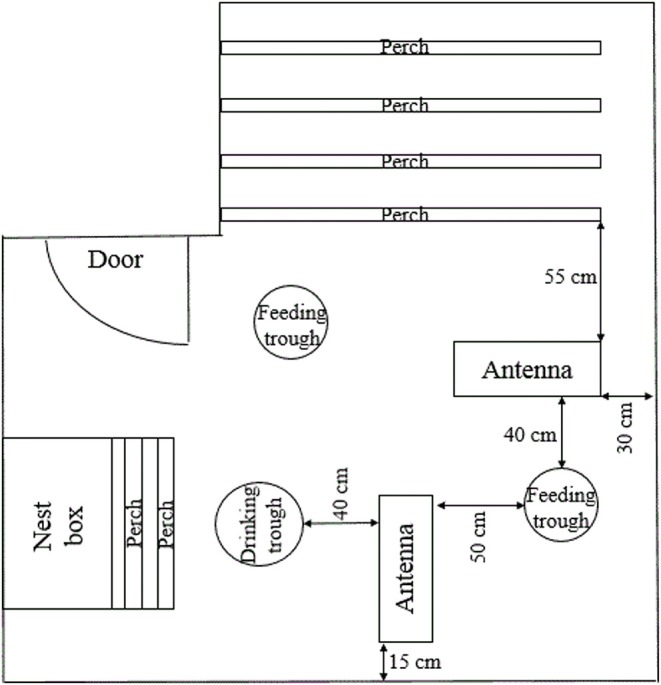
Antennae location. Schematic, not to scale representation of the pen, demonstrating the location of both antennae in relation to the elements of the housing equipment.

Locomotor activity was recorded at the end of each sampling period by the use of an electronic transponder system (Gantner Pigeon Systems GmbH, Schruns, Austria) as described by others (46–48). All hens were fitted with an electronic transponder that was attached to the right leg with a plastic case and cable straps and which was individually identifiable by the antennas. Two antennas (76 × 29.5 × 3 cm) were placed on the floor of each pen (Figure 2). One antenna was placed between the perches and one of the two feeding troughs (distance to the closest perch: 55 cm, distance to the feeding trough: 40 cm). The other antenna was placed at right angles with the first one between the same feeding trough and the drinking trough (distance to the feeding trough: 50 cm, distance to the drinking trough: 40 cm). Within a 15 cm range of an antenna, transponders and thereby hens were individually registered and hen identity, antenna location, date and time of registration were recorded and stored as ASCII files. Analysis (SAS^®^ 9.4, SAS Institute Inc., Cary, NC) included the lighting period of 10 (sampling period 7), 13 (sampling period 6), or 14 (sampling periods 1 to 5) days, respectively. Since lighting period differed between sampling periods, locomotor activity was measured as the mean number of antenna crossings/h for each sampling period resulting in one value per hen and sampling period.

### Statistical Analysis

Statistical analysis was performed in R 3.5.2 (49). In order to adequately reflect dependencies in the experimental design (nesting, repeated measurements), generalized linear mixed-effects models were used to evaluate the numerical outcome variables body weight, POD (log transformed), radiographic density, estradiol-17ß plasma concentration (log transformed), and locomotor activity (log transformed) with the lme method from the nlme package (50). In each of these models, layer line (factor with two levels: G11 and WLA), treatment (factor with four levels: C, E, S, and SE), sampling period (factor with seven levels: 1–7), and all two-way interactions as well as the three-way interaction were included as fixed effects. Hen nested in pen was included as random effect. To avoid multiple hypothesis testing, no model simplification was performed (51). Significant *p*-values were obtained from examination of the full model and the test statistic for the full model is presented for each outcome variable in the results section. For analysis of POD, only hens with a keel bone deviation were included in the analysis. Since the model including all seven levels of the fixed effect sampling period was overspecified due to only a few hens having deviations in sampling periods 1 (4 hens) and 2 (31 hens), only sampling periods 3–7 were included in the analysis of POD. Results of all numerical outcome variables are described using the model estimates.

The effect of layer line and treatment on the occurrence of fractures and deviations (yes/no) was assessed with a survival analysis using the coxph method from the survival package (52, 53). Consequently, “survival” was equivalent with an intact keel bone, i.e., no occurrence of fractures or deviations, respectively. Only hens that lived until the end of the experiment and whose keel bone could consistently be assessed for the occurrence of fractures or deviations were included in the respective survival analysis. Some radiographs could be assessed for occurrence of deviations but not for occurrence of fractures because the legs of the hen overlapped with the caudal part of the keel bone where most of the fractures occurred while deviations were usually present in the middle or cranial part of the keel bone. Thus, number of animals varied between analysis of deviations on the one hand and fractures on the other hand. In both models, layer line (factor with two levels: G11 and WLA) and treatment (factor with four levels: C, E, S, and SE) were included as fixed effects. Pen was included as random effect. For analysis of keel bone deviations, the interaction between layer line and treatment group was included in the model, which was not possible for analysis of keel bone fractures due to model overspecification.

All model assumptions were verified using graphical analysis of residuals.

## Results

### Ultrasonography of Ovaries

All control hens (group C) and hens treated only with estradiol-17ß (group E) showed ovarian follicles in all examinations. One of the hens treated with deslorelin acetate (group S) of layer line WLA still showed ovarian follicles after implantation and, thus, was excluded from statistical analysis. None of the hens treated with both implants (group SE) showed ovarian follicles after implantation.

### Body Weight

**Figure 3:**
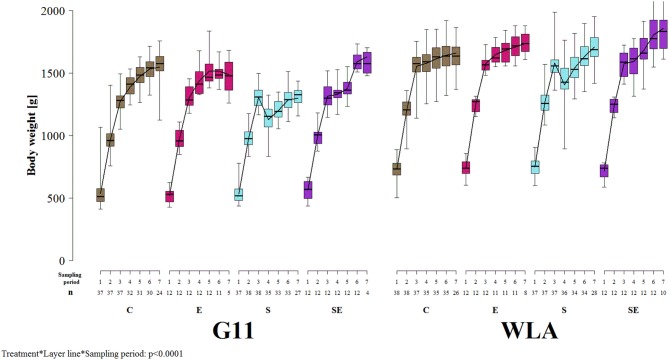
Body weight. Each boxplot represents the median, first and third quartile, and minimum and maximum of the body weight of a specific treatment group and layer line within a certain sampling period. The black lines represent the model estimates. Numbers of hens that were included in the analysis are given under each sampling period. C, control hens (egg laying); E, hens treated with estradiol-17ß (egg laying); S, hens treated with deslorelin acetate (non-egg laying); SE, hens treated with deslorelin acetate and estradiol-17ß (non-egg laying); G11, low performing layer line; WLA, high performing layer line; Sampling period 1 = 12th−13th week of age, 2 = 19th−20th week of age, 3 = 26th−27th week of age, 4 = 32nd−33rd week of age, 5 = 39th−40th week of age, 6 = 49th−50th week of age, 7 = 60th−61st week of age.

Body weight was significantly influenced by the three-way interaction between treatment, layer line, and sampling period [*F*_(18, 1021)_ = 7.1, *p* < 0.0001; Figure 3]. In all treatment groups of both layer lines, body weight increased until sampling period 3. In contrast to the other treatment groups, body weight of group S of both layer lines decreased between sampling periods 3 and 4 and increased after sampling period 4 again. Body weight was lower in layer line G11 compared to WLA throughout the entire experimental period (groups E, S, and SE) or until sampling period 6 (group C), respectively.

### Radiographic Examination of the Keel Bone

#### Fractures

**Figure 4:**
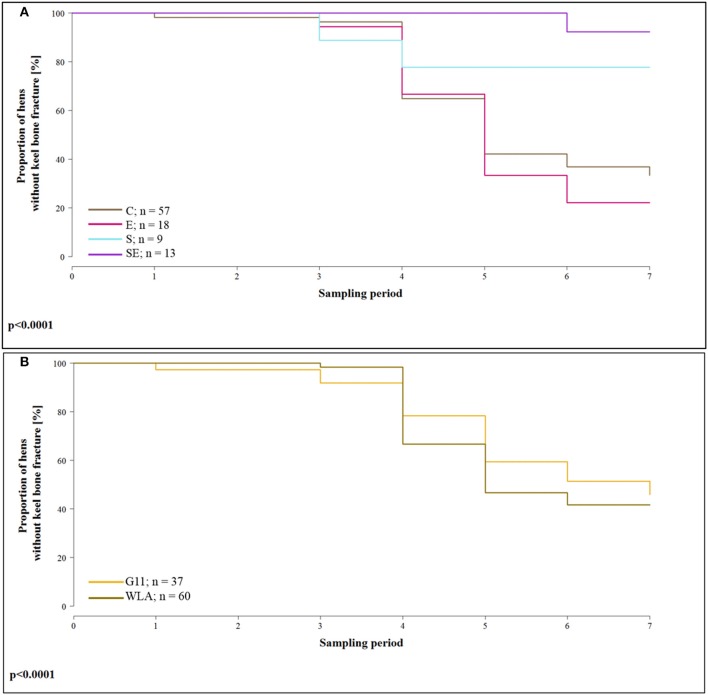
Survival analysis of keel bone fractures. Note that only hens that lived until the end of the study and of which all seven radiographs could be evaluated for fractures were included in the analysis. **(A)** Proportion of hens without keel bone fractures of the four different treatment groups for all seven sampling periods. **(B)** Proportion of hens without keel bone fractures of the two different layer lines for all seven sampling periods. These data include all treatment groups. C, control hens (egg laying); E, hens treated with estradiol-17ß (egg laying); S, hens treated with deslorelin acetate (non-egg laying)l; SE, hens treated with deslorelin acetate and estradiol-17ß (non-egg laying); G11, low performing layer line; WLA, high performing layer line; Sampling period 1 = 13th week of age, 2 = 20th week of age, 3 = 27th week of age, 4 = 33rd week of age, 5 = 40th week of age, 6 = 50th week of age, 7 = 61st week of age.

The proportion of hens whose keel bone was not fractured throughout the experimental period was higher in group S (hazard ratio: 0.20, 95% confidence interval (CI) [0.11; 0.35]; i.e., risk of keel bone fracture reduced by 80%) and in group SE [hazard ratio: 0.06, CI [0.006; 0.69]; i.e., risk reduced by 94%] compared to group C. In contrast, the proportion of hens without fractured keel bone was lower in group E [hazard ratio: 1.17, CI [0.52; 2.60]; i.e., risk increased by 17%) compared to group C (Figure 4A). In layer line WLA, the proportion of hens without fractured keel bone was lower compared to layer line G11 [hazard ratio: 1.66, CI [1.32; 2.10], i.e., risk of keel bone fracture increased by 66%; Figure 4B; treatment + layer line: Wald test: $\chi_{4}^{2}$ = 134; *p* < 0.0001]. This analysis only included hens that lived until the end of the study and in which all radiographs could be assessed for the presence of fractures.

In addition, Table 2 presents the prevalence of keel bone fractures for each sampling period including all hens that were radiographed and whose radiograph could be evaluated in the respective sampling period.

**Table 2:** Prevalence of keel bone fractures of all radiographed hens.

**Treatment group**	**Sampling period 1 13th woa**	**Sampling period 2 20th woa**	**Sampling period 3 27th woa**	**Sampling period 4 33rd woa**	**Sampling period 5 40th woa**	**Sampling period 6 50th woa**	**Sampling period 7 61st woa**
**Layer line G11**							
C	0% (0/35)	0% (0 /33)	0% (0/24)	12.9% (4/31)	28.57% (8/28)	35.71% (10/28)	43.48% (10/23)
E	0% (0/11)	0% (0/12)	10% (1/10)	27.27% (3/11)	50% (6/12)	60% (6/10)	33.33% (1/3)
S	0% (0/37)	0% (0/27)	0% (0/28)	0% (0/3)	0% (0/3)	n.a. (0/0)	n.a. (0/0)
SE	0% (0/12)	0% (0/10)	0% (0/11)	0% (0/5)	0% (0/2)	25% (1/4)	0% (0/3)
**Layer line WLA**							
C	0% (0/38)	0% (0/36)	0% (0/36)	45.45% (15/33)	63.89% (23/36)	74.29% (26/35)	76.92% (20/26)
E	0% (0/12)	0% (0/12)	0% (0/12)	27.27% (3/11)	54.55% (6/11)	63.64% (7/11)	62.5% (5/8)
S	0% (0/36)	0% (0/30)	2.86% (1/35)	8.33% (1/12)	0% (0/9)	0% (0/9)	0% (0/8)
SE	0% (0/11)	0% (0/12)	0% (0/11)	0% (0/10)	0% (0/10)	0% (0/8)	0% (0/9)

*Each cell indicates the prevalence of keel bone fractures for a specific layer line and treatment group in a specific sampling period. Note that in contrast to Figure 3, also hens that died before the end of the study or in which only part of the radiographs could be evaluated for the presence of fractures were included in the calculation of fracture prevalence. The numbers in brackets in each cell indicate the number of hens with a keel bone fracture against the number of hens that were radiographed and whose radiograph could be evaluated for a specific treatment group and sampling period. woa, week of age; n.a., No radiograph of this group could be evaluated in this sampling period; C, control hens (egg laying); E, hens treated with estradiol-17ß (egg laying); S, hens treated with deslorelin acetate (non-egg laying); SE, hens treated with deslorelin acetate and estradiol-17ß (non-egg laying); G11, low performing layer line; WLA, high performing layer line*.

#### Deviations

**Figure 5:**
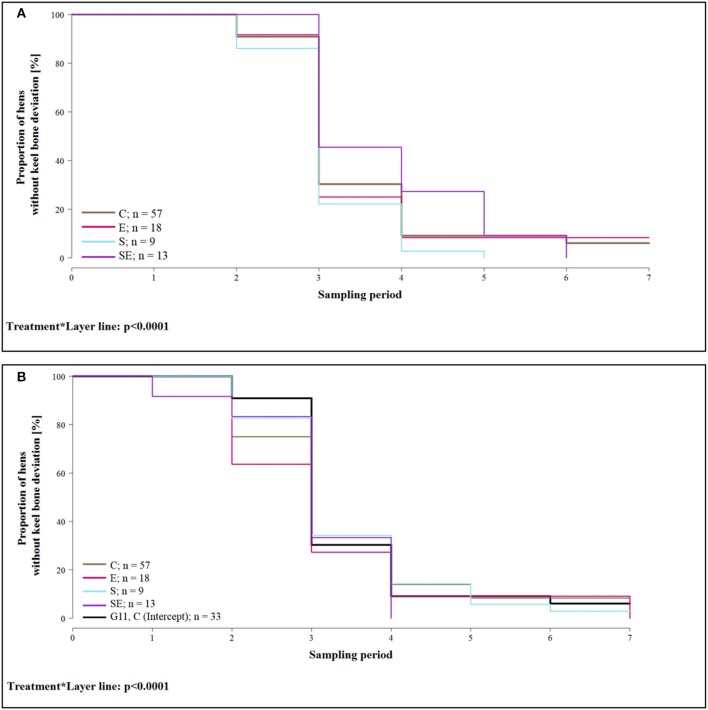
Survival analysis of keel bone deviations. Note that only hens that lived until the end of the study were included in the analysis. **(A)** Proportion of hens without keel bone deviations of layer line G11 for the four different treatment groups and all seven sampling periods. **(B)** Proportion of hens without keel bone deviations of layer line WLA for the four different treatment groups and all seven sampling periods. For comparison, the intercept (control hens of layer line G11) is also included in the graph (black, bold line). C, control hens (egg laying); E, hens treated with estradiol-17ß (egg laying); S, hens treated with deslorelin acetate (non-egg laying); SE, hens treated with deslorelin acetate and estradiol-17ß (non-egg laying); G11, low performing layer line; WLA, high performing layer line; Sampling period 1 = 13th week of age, 2 = 20th week of age, 3 = 27th week of age, 4 = 33rd week of age, 5 = 40th week of age, 6 = 50th week of age, 7 = 61st week of age.

The proportion of hens whose keel bone was not deviated throughout the experimental period was lower in group E [hazard ratio: 1.03, CI [0.58; 1.84]; i.e., risk of keel bone deviation increased by 3%] and group S [hazard ratio: 1.37, CI [1.26; 1.49]; i.e., risk increased by 37%] of layer line G11 compared to group C of layer line G11 (= reference group / intercept). In contrast, the proportion of hens without deviated keel bone was higher in group SE of layer line G11 compared to group C of layer line G11 [hazard ratio: 0.78, CI [0.53; 1.16]; i.e., risk reduced by 22%]. Proportion of hens without deviation was higher in groups C (hazard ratio: 0.98, CI [0.67; 1.43]; i.e., risk reduced by 2%) and S [hazard ratio: 0.75, CI [0.65; 0.87]; i.e., risk reduced by 25%] of layer line WLA but lower in groups E [hazard ratio: 1.25, CI [0.67; 2.34]; i.e., risk increased by 25%] and SE [hazard ratio: 1.65, CI [0.70; 3.89]; i.e., risk increased by 65%] of layer line WLA compared to group C of layer line G11 (treatment^*^layer line: Wald test: $\chi_{7}^{2}$ = 990.5; *p* < 0.0001; Figures 5A,B). This analysis only included hens that lived until the end of the study. For purposes of clarity, survival analysis is presented in two separate graphs for the layer lines in spite of the significant interaction between layer line and treatment. The reference group (intercept), i.e., treatment group C of layer line G11, has been added to the graph of layer line WLA so that comparisons can be made between the groups of WLA and the reference group.

**Figure 6:**
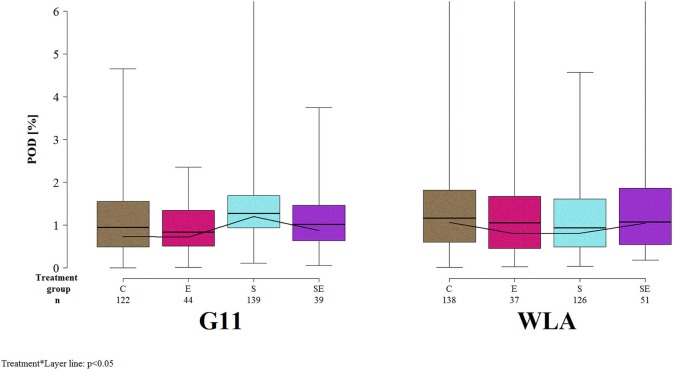
Severity of keel bone deviations. Each boxplot represents the median, first and third quartile, and minimum and maximum of the proportion of the deviated keel bone area (POD) of a specific treatment group and layer line within a certain sampling period. The black lines represent the model estimates. Numbers of hens that were included in the analysis are given under each treatment group. C, control hens (egg laying); E, hens treated with estradiol-17ß (egg laying); S, hens treated with deslorelin acetate (non-egg laying); SE, hens treated with deslorelin acetate and estradiol-17ß (non-egg laying); G11, low performing layer line; WLA, high performing layer line; Sampling period 1 = 13th week of age, 2 = 20th week of age, 3 = 27th week of age, 4 = 33rd week of age, 5 = 40th week of age, 6 = 50th week of age, 7 = 61st week of age.

The proportion of the deviated keel bone area (POD) was not significantly affected by the three-way interaction between layer line, treatment, and sampling period (*F*_(12, 3486)_ = 1.06, *p* = 0.39] but by the two-way interaction between layer line and treatment [*F*_(3, 168)_ = 3.3, *p* = 0.0219; Figure 6]. Within layer line G11, POD was higher in group S compared to all other treatment groups. In contrast, within layer line WLA, POD was lower in groups E and S compared to groups C and SE. Within groups C, E, and SE, POD was slightly higher in WLA compared to G11. Within group S, POD was higher in layer line G11 compared to WLA.

#### Radiographic Density

**Figure 7:**
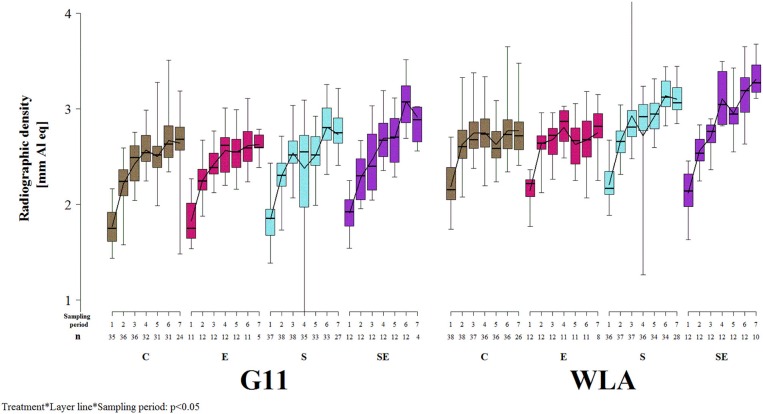
Radiographic density of the keel bone. Each boxplot represents the median, first and third quartile, and minimum and maximum of the radiographic density of a specific treatment group and layer line within a certain sampling period. The black lines represent the model estimates. Numbers of hens that were included in the analysis are given under each sampling period. C, control hens (egg laying); E, hens treated with estradiol-17ß (egg laying); S, hens treated with deslorelin acetate (non-egg laying); SE, hens treated with deslorelin acetate and estradiol-17ß (non-egg laying); G11, low performing layer line; WLA, high performing layer line; Sampling period 1 = 13th week of age, 2 = 20th week of age, 3 = 27th week of age, 4 = 33rd week of age, 5 = 40th week of age, 6 = 50th week of age, 7 = 61st week of age.

Radiographic density of the keel bone was significantly influenced by the three-way interaction between treatment, layer line, and sampling period (*F*_(18, 1019)_ = 1.91, *p* = 0.0123; Figure 7]. Radiographic density increased at the beginning of the study in all treatment groups of both layer lines. This increase leveled off in groups C and E of both layer lines after sampling period 4. In contrast, radiographic density decreased between sampling periods 3 and 4 but markedly increased thereafter in group S of both layer lines while it steadily increased throughout the study in group SE of both layer lines. Thus, treatment groups S and SE reached higher radiographic density values compared to groups C and E in both layer lines toward the end of the study. Furthermore, group SE showed a higher radiographic density compared to group S from sampling period 4 onwards, which was more pronounced in layer line G11. WLA showed a higher radiographic density compared to G11 until sampling period 4 within groups C and E and throughout the study within groups S and SE, respectively.

### Estradiol-17ß Plasma Concentration

**Figure 8:**
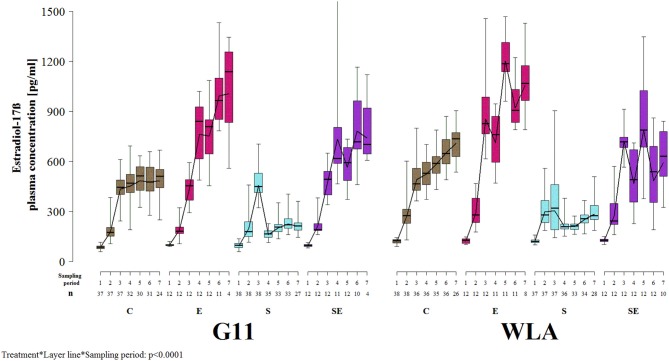
Estradiol-17ß plasma concentration. Each boxplot represents the median, first and third quartile, and minimum and maximum of the estradiol-17ß plasma concentration of a specific treatment group and layer line within a certain sampling period. The black lines represent the model estimates. Numbers of hens that were included in the analysis are given under each sampling period. C, control hens (egg laying); E, hens treated with estradiol-17ß (egg laying); S, hens treated with deslorelin acetate (non-egg laying); SE, hens treated with deslorelin acetate and estradiol-17ß (non-egg laying); G11, low performing layer line; WLA = high performing layer line; Sampling period 1 = 12th−13th week of age, 2 = 19th−20th week of age, 3 = 26th−27th week of age, 4 = 32nd−33rd week of age, 5 = 39th−40th week of age, 6 = 49th−50th week of age, 7 = 60th−61st week of age.

Estradiol-17ß plasma concentration was significantly influenced by the three-way interaction between layer line, treatment, and sampling period (*F*_8, 1018_ = 15.3, *p* < 0.0001; Figure 8). In all treatment groups of both layer lines, estradiol-17ß concentration increased until sampling period 3. In group C of layer line G11, it did not increase or decrease beyond sampling period 3, while it increased throughout the entire experimental period in group C of layer line WLA. Thus, control hens of layer line WLA reached higher estradiol-17ß plasma concentrations than control hens of layer line G11. In group E of both layer lines, estradiol-17ß plasma concentration increased throughout the experiment and reached higher values compared to group C, although concentration varied a lot between sampling periods in group E of layer line WLA which was not the case in group E of layer line G11. In group S of both layer lines, estradiol-17ß concentration decreased between sampling periods 3 and 4 and stayed at a low level, although increasing again toward the end of the experiment, especially in group S of layer line WLA. In group SE of both layer lines, estradiol-17ß plasma concentrations varied a lot between sampling periods but reached higher values compared to group S. The variation between sampling periods was more pronounced in group SE of layer line WLA compared to group SE of layer line G11. Within layer line G11, estradiol-17ß plasma concentration was highest in group E, followed by group SE, C, and S from sampling period 4 onwards. Within layer line WLA, estradiol-17ß plasma concentration was highest in group E, followed by groups C and SE which showed comparable values, and lowest in group S from sampling period 3 onwards.

### Locomotor Activity

Locomotor activity was not significantly affected by the three-way interaction between layer line, treatment, and sampling period [*F*_(18, 984)_ = 1.30, *p* = 0.18] but by two two-way interactions.

**Figure 9:**
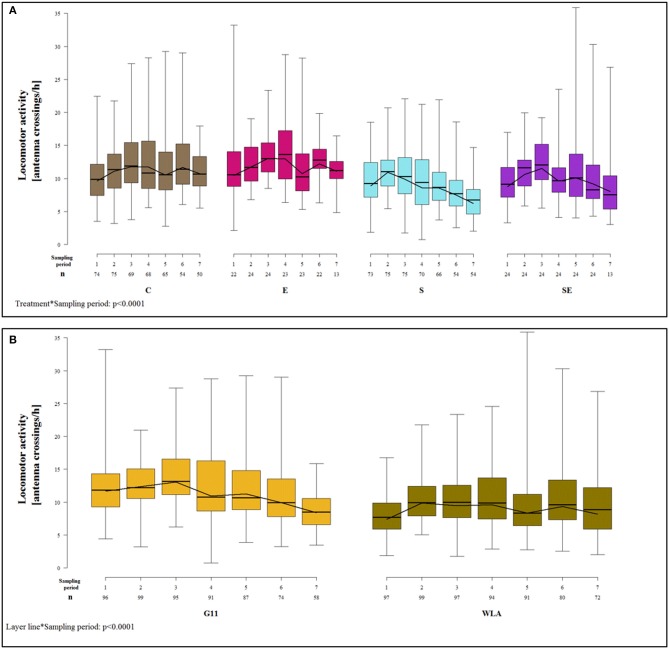
Locomotor activity. Each boxplot represents the median, first and third quartile, and minimum and maximum of the locomotor activity of **(A)** a specific treatment group within a certain sampling period. The black lines represent the model estimates. Numbers of hens that were included in the analysis are given under each sampling period. **(B)** a specific layer line within a certain sampling period. The black lines represent the model estimates. Numbers of hens that were included in the analysis are given under each sampling period. C, control hens (egg laying); E, hens treated with estradiol-17ß (egg laying); S, hens treated with deslorelin acetate (non-egg laying); SE, hens treated with deslorelin acetate and estradiol-17ß (non-egg laying); G11, low performing layer line; WLA, high performing layer line; Sampling period 1 = 14th−15th week of age, 2 = 21st−22nd week of age, 3 = 28th−29th week of age, 4 = 34th−35th week of age, 5 = 41st−42nd week of age, 6 = 51st−52nd week of age, 7 = 61st−62nd week of age.

The treatment groups showed very different patterns of locomotor activity over time. In groups C and E, locomotor activity did not vary a lot between sampling periods. In contrast, groups S and SE showed a steady decrease in locomotor activity from sampling period 2 (group S) or sampling period 3 (group SE) onwards, respectively. Thus, toward the end of the study, locomotor activity was higher in groups C and E compared to groups S and SE [sampling period^*^treatment: *F*_(18, 984)_ = 4.16, *p* < 0.0001; Figure 9A].

The patterns of locomotor activity over time also differed between layer lines. In G11, locomotor activity was higher in the first three sampling periods compared to the last four. In WLA, locomotor activity was lower in sampling periods 1, 5, and 7 compared to the other sampling periods. Locomotor activity differed between layer lines at the beginning of the study (sampling periods 1–3) with G11 being more active compared to WLA. However, this difference leveled off from sampling period 4 onwards [sampling period^*^layer line: *F*_(6, 984)_ = 9.45, *p* < 0.0001; Figure 9B].

## Discussion

### Egg Production and Keel Bone Damage

Our results clearly indicate that there is an association between egg production and keel bone fractures as well as radiographic density of the keel bone but that no association seems to exist between egg production and keel bone deviations.

As hypothesized, the risk of keel bone fracture was markedly lower in non-egg laying compared to egg laying hens. In comparison to group C, fracture risk was decreased by 80% in hens of group S and by 94% in hens of group SE. This is consistent with a previous study in which egg laying hens showed a significantly higher prevalence of keel bone fractures (up to 40%) compared to non-egg laying hens (0% throughout) (28). These findings suggest that egg production makes the keel bone very susceptible to fractures. It is crucial to find out which are the mechanisms behind the different risk of keel bone fracture in non-egg laying and egg laying hens. It is known that fracture risk and bone strength are influenced by material properties such as degree of mineralization, mineral composition, crystallinity, collagen characteristics, and osteocyte viability and by structural properties such as thickness and porosity of the cortex as well as thickness and connectivity of the trabeculae (54, 55). In order to find solutions against keel bone fractures, it seems essential to find out whether the keel bone of egg laying hens differs from that of non-egg laying hens in any of these characteristics. On the one hand, it is possible that egg laying hens resorbed more calcium from the bones for the egg shell, leading to poorer bone quality, or that energy balance differed between egg laying and non-egg laying hens, resulting in increased resource availability for anabolic processes, including bone growth, in non-egg laying hens. On the other hand, it is also possible that deslorelin acetate had a direct effect on bone characteristics, independently of egg production. However, the potential direct effect of this drug on bone health does not seem to be likely to be positive as studies investigating the effect of GnRH agonists on bone traits in humans found lower bone densities in treated compared to non-treated persons (56, 57). Nevertheless, further studies comparing egg-laying with non-egg laying hens but suppressing egg production by other means would be helpful to further assess the role of egg production in keel bone damage.

One characteristic of the keel bone that differed between egg laying and non-egg laying hens in the present study was the radiographic density. At the end of the study, it reached higher values in non-egg laying hens (groups S and SE) compared to egg laying hens (groups C and E). The radiographic density reflects bone mineral density (BMD) (45, 58). Thus, BMD of the keel bone seemed to be higher in non-egg laying compared to egg laying hens toward the end of the study. This may be an underlying cause of the higher fracture prevalence in egg laying hens. A relationship between radiographic density or BMD and bone strength in laying hens has been shown in other studies (35, 45). Furthermore, Toscano et al. (59) found that increased BMD of the keel bone decreased the likelihood of an experimental keel bone fracture. In humans, it has been shown that a model including the change in BMD is more suitable to estimate the risk of fractures compared to a model that only includes baseline BMD (60). In the current study, radiographic density decreased between sampling periods 4 and 5, i.e., between the 33rd and the 40th week of age, in groups C and E of layer line WLA and, less pronounced, of layer line G11. This was also the time when fracture prevalence increased the most. Thus, the results concerning radiographic density and fracture risk in egg laying compared to non-egg laying hens indicate that radiographic density of the keel bone as assessed in the current study seems to be a suitable approach to predict keel bone strength and that keel bone fractures seem indeed to be associated with changes in BMD throughout the laying cycle. However, this is in contrast to findings about radiographic density and fracture risk between the different layer lines where the high performing layer line showed a higher radiographic density but also a higher risk of fracture compared to the low performing layer line as discussed in section Layer Line and Keel Bone Damage. Radiographic density and BMD can be influenced by bone mass and the degree of mineralization. Thus, one of or both these characteristics may differ between the keel bone of egg laying and non-egg laying hens. It has been suggested that lack of bone mass is an underlying cause of keel bone fractures (13). It has also been suggested that this loss in bone mass takes place because during the laying period, only medullary bone and no structural bone is formed while osteoclastic resorption of structural bone continues and that structural bone formation only recommences when the hen goes out of lay (27). While this cycle of bone loss and regeneration seems to allow to maintain good bone quality in female birds that lay eggs in clutches followed by incubation, commercial laying hens have been selected to remain in a continuously reproductive condition and their bones do not seem to have time to regenerate (27). While our data suggest that changes in bone mass may indeed contribute to the high prevalence of keel bone fractures, they do not allow to support the hypothesis that there is a continuous loss of bone mass during lay. Radiographic density only decreased between sampling periods 4 and 5 in egg laying hens but increased again thereafter. This may indicate that no loss of bone mass occurred after the 40th week of age and that the bones even recovered from the temporary loss of bone mass. It is important to note that the hypothesis about a continuous loss of bone mass was constructed based on studies that took place when the majority of hens was still housed in single cages (27) while the hens of the present study were kept in a floor housing system. It is possible that in caged hens in which movement is restricted, bone mass does indeed continuously decrease during lay while this is not the case if hens are kept in a housing system which allows for load-bearing movement. However, radiographic density as assessed in the present study includes structural and medullary bone. Thus, it is also possible that radiographic density was kept at a high level due to a high amount of medullary rather than structural bone in egg laying hens. While this type of bone shows a high degree of mineralization, it is weaker than structural bone, and thus, does not contribute to bone strength to the same extent as structural bone (27). This would explain why in egg laying hens radiographic density did not continuously decrease while fracture prevalence increased throughout the study. It is also important to mention that radiographic density as measured in the present study may also have been influenced by other factors, such as the breast muscles, and not by BMD alone. Thus, further examinations that allow for assessing BMD into more detail and for distinguishing between structural and medullary bone are required in order to further analyze bone structure of egg laying and non-egg laying hens. These could include radiographic density assessment of isolated keel bones, chemical analyses or histological analyses.

Another factor that differed between treatment groups is the body weight. Hens of group C were heavier compared to hens of group S from sampling period 4 onwards in layer line G11 and in sampling periods 4 to 6 in layer line WLA. This is consistent with a previous study in which control hens were also heavier compared to hens treated with deslorelin acetate (28). In that previous study, we assumed that the higher body weight in egg laying compared to non-egg laying hens could be the result of the increased weight of the ovary and oviduct alone. However, hens of group SE of the current study were as heavy or even heavier than hens of group C although they did not show ovarian follicles and, thus, the weight of their ovary and oviduct is assumed to have been as low as that of group S. This indicates that estradiol-17ß seems to have an influence on body weight, but the mechanisms remain unknown to us. Body weight is discussed to affect the occurrence of keel bone fractures and deviations. On the one hand, it is assumed that higher body weight may increase the risk of keel bone fracture due to greater collision energies when colliding with a perch (5, 15). On the other hand, it is possible that a higher breast muscle mass, which also increases body weight, may have a protective effect on the keel bone because low breast muscle mass leaves the keel vulnerable to fracture (13). Furthermore, increased body weight has been found to be associated with increased bone strength in laying hens, probably due to increased mechanical loading on the bone (61). However, in the current study, it seems unlikely that differences in body weight development have influenced fracture risk due to the similar body weight in hens of groups C and SE but the much higher risk of fracture in group C compared to group SE.

Contrary to our hypothesis and in contrast to fractures, neither the risk of keel bone deviation nor the severity of the present deviations, i.e., POD, were clearly influenced by egg production. Although hens of group S showed a lower POD compared to control hens within layer line WLA, this effect was reverse in layer line G11 and, within this layer line, group S also showed a higher risk of deviation compared to group C. This indicates that keel bone fractures and keel bone deviations are two independent phenomena caused by different factors. This assumption is in accordance with findings of a previous study in which deviations were more severe in cage housed hens while fractures were more frequent in their floor housed siblings, indicating different risk factors for these two types of damage (19). Results of that previous study suggest that deviations are mainly caused by the pressure of the perch on the keel bone. Likewise, in the present study, all pens were equipped with perches and, thus, deviations may have been caused by the pressure on the keel bone while perching. This is in accordance with findings that in a perching laying hen, the peak force is ~5 times higher on the keel bone compared to a single foot pad (62), indicating that most of the hen's weight is supported by the keel bone. Interestingly, suppressed egg production did not protect the keel bone against this kind of keel bone damage. In contrast, hens of group S even showed a higher risk of keel bone deviations compared to group C within layer line G11. It is possible that this was caused by a higher perch use in hens of this group but this has not been assessed in this study. Results of the present study are not in accordance with findings of another previous study in which POD was significantly higher in egg laying control compared to hens that were treated with a deslorelin acetate implant after the onset of lay (28). This may be explained by different genetics in both studies. In the previous study, we used the hybrid Lohmann Selected Leghorn (LSL) while in the current study, the purebred layer lines WLA and G11 were used. Furthermore, the influence of the treatment on the severity of deviations was also analyzed differently in both studies. While in the previous study, all hens were included in the statistical analysis of POD (28), only hens that actually showed a keel bone deviation were included in the statistical analysis of POD in the present study. This may also explain the different findings of the two studies.

### Estradiol-17ß and Keel Bone Damage

Treatment with estradiol-17ß implants had a strong effect on estradiol-17ß plasma concentration but its impact on keel bone health differed between egg laying and non-egg laying hens.

Estradiol-17ß plasma concentration differed between treatment groups and confirmed the effectiveness of the administered implants. The concentration of this gonadal steroid was decreased by administration of the sustained release deslorelin acetate implant (i.e., in group S). This finding is consistent with findings about estradiol-17ß concentrations after administration of a sustained release deslorelin acetate implant in laying hens (28), Japanese quail (63), and ferrets (64). Administration of a subcutaneous implant containing estradiol-17ß in hens treated with deslorelin acetate (i.e., group SE) increased the plasma concentration of this hormone to a concentration which was comparable to that found in control hens (layer line WLA) or even higher (layer line G11). Similarly, administration of an estradiol-17ß implant in laying hens without deslorelin acetate (i.e., group E) increased the hormone concentration above the concentration which was found in control hens (group C). Thus, after administration of the implants, estradiol-17ß plasma concentration was highest in group E, followed by groups SE and C, and lowest in group S.

However, no clear effect of these different estradiol-17ß plasma concentrations on keel bone health was found. Concerning non-egg laying hens, hens of group SE, which showed a similar or even higher estradiol-17ß plasma concentration compared to control hens, were at a lower fracture risk compared to hens of group C. Interestingly and contrary to our hypothesis, hens of group SE also were at a lower risk of keel bone fracture compared to hens of group S which showed a much lower estradiol-17ß plasma concentration. Furthermore, radiographic density was higher in group SE from sampling period 4 onwards and, although only within layer line G11, risk of keel bone deviation and POD were higher in group S compared to group SE. Thus, treatment with an estradiol-17ß implant did not diminish the positive effect of deslorelin acetate on keel bone health by increasing estradiol-17ß plasma concentrations. In contrast, both implants seemed to have a synergistic, positive effect on keel bone health. This clearly shows that the lower risk of keel bone fracture in non-egg laying compared to egg laying hens was not caused by lower estradiol-17ß plasma concentrations in non-egg laying hens and that the positive influence of deslorelin acetate on the keel bone was not related to the decrease of estradiol-17ß caused by this implant. The results of groups S and SE also suggest that estradiol-17ß may have a protective effect on the keel bone if no egg production occurs. Within egg laying hens, the risk of keel bone fracture was moderately increased in hens treated with only estradiol-17ß (group E) compared to control hens (group C). In contrast, radiographic density of the keel bone did not differ between groups E and C and no clear relationship between treatment with estradiol-17ß and keel bone deviations was found either. Thus, our findings indicate that estradiol-17ß plasma concentrations above physiological (i.e., found in control hens) concentrations may lead to a higher keel bone fracture risk in egg laying hens but do not fully support findings by other authors about a large negative influence of estradiol on bone health in chickens. Urist and Deutsch (32) found that treating laying hens with exogenous estradiol led to a thinner cortex and lower breaking strength of the long bones and assumed that exogenous estradiol accentuated osteoporosis. Reduced bone strength and defects in the cortical bone were also found after treatment with exogenous estradiol in roosters (32) and capons (30). The fact that exogenous estradiol had a large influence on bone health in these studies but only a moderate influence on fracture risk in egg-laying hens in the present study may be explained by different amounts of exogenous estradiol. The hens in the study by Urist and Deutsch (32) received 100 mg exogenous estradiol per week for 4 weeks while the administered implant in the current study contained 75 mg estradiol-17ß and lasted for 12 weeks. Thus, it is possible that the concentration of the administered estradiol-17ß was too low to show a large effect on the skeleton. Furthermore, the other authors examined long bones while we assessed the keel bone, which may also explain different findings. Taken together, our results indicate that estradiol-17ß has a different effect on keel bone health in egg laying compared to non-egg laying hens and suggest that detailed analyses of the mechanisms behind these effects may help to better understand the causes of keel bone damage in laying hens.

### Layer Line and Keel Bone Damage

Similar to egg production, layer line had an influence on keel bone fractures but not on keel bone deviations.

The high performing layer line WLA showed a higher risk of keel bone fracture compared to the low performing layer line G11. This is consistent with a previous study in which we also found more keel bone fractures in a high compared to a low performing layer line (19). In accordance with these findings, Habig et al. (65), who worked with the same layer lines, found a higher breaking strength and bone mineral density of long bones in the low compared to the high performing layer lines. Furthermore, Candelotto et al. (24) found a lower number of experimental keel bone fractures in an experimental line that descended from a dam line which had not been selected for any breeding goal for several years and a sire line which had been bred for dual egg and meat production compared to the lines that had been selected for high productivity. In a study by Hocking et al. (33), a higher radiographic density of the keel bone as well as a higher breaking strength of the humerus and tibiotarsus were found in traditional layer lines with a low laying performance compared to commercial layer lines with a high laying performance (33). The results of all studies together may indicate that selection for high laying performance has led to poor bone health and a higher risk of keel bone fractures. However, interestingly, WLA, although having a higher risk of keel bone fracture, had a higher radiographic density compared to G11 in the present study. This was true for the whole experimental period within groups S and SE and until sampling period 4 within groups C and E. There are different possible explanations for this phenomenon. On the one hand, it is possible that other differences between the layer lines that were not subject to the present study played a more important role in the etiology of keel bone fractures compared to BMD, i.e., bone mass and degree of mineralization. These could be a number of other differences in bone characteristics such as crystallinity, collagen characteristics, osteocyte viability, or thickness and connectivity of the trabeculae (54, 55). There could also be behavioral differences, e.g., differences in perch use and in motor skills, i.e., flight and 3D-movement skills, between the layer lines that led to a higher fracture risk in WLA. However, on the other hand, it is also possible that radiographic density as assessed in the present study does not allow to readily draw conclusions about the degree of mineralization and amount of structural bone. As mentioned above, the higher radiographic density may also reflect a higher amount of medullary bone in WLA compared to G11 which is weaker than structural bone (27). Furthermore, also factors that are not directly related to the keel bone may have influenced the radiographic density in this study. WLA hens were heavier compared to G11 hens. Thus, it is possible that WLA had a higher breast muscle mass which may have led to the higher radiographic density. Again, further analyses of the BMD and the structure of the keel bone would be required to assess the relevance of the higher radiographic density in WLA compared to G11 in the present study. Differences in fracture risk between the two layer lines cannot directly be linked to the different laying performance as only one high and one low performing layer line have been examined that also differ in other characteristics. To name only one, age at onset of lay differed between layer lines of the present and at least some of the other mentioned studies that found differences in keel bone health between high and low performing layer lines (33, 65). High performing hens were younger when they started to lay eggs compared to low performing hens. Thus, the early onset of lay may additionally have influenced keel bone health. Gebhardt-Henrich and Fröhlich (66) found a negative correlation between the age of hens when laying their first egg and the probability of keel bone fracture presence at depopulation. Thus, it may be possible to decrease the prevalence of keel bone fractures by protracting the onset of lay in commercial laying hens, for example with the help of the lighting regime. However, more studies are required to assess the possible role of the early onset of lay in the etiology of keel bone fractures.

Contrary to our hypothesis, control hens of the high performing layer line WLA showed a lower risk of keel bone deviation compared to control hens of the low performing layer line G11. However, this difference was only very marginal (risk reduced by 2%) and, thus, the biological relevance of this finding is debatable. Concerning severity of deviations, within treatment groups C, E, and SE, POD was slightly higher in WLA compared to G11 while the opposite was the case within group S. This is partly in contrast to and partly consistent with findings of a previous study (19). In that study, prevalence of deviations was higher in a high performing compared to a low performing brown layer line and higher in WLA compared to G11 but not compared to another low performing white layer line (R11). Moreover, differences between the layer lines in terms of POD were only found for hens housed in cages, where it increased in the high performing lines and in R11 but not in the other two low performing lines. In hens housed in a floor system, no differences were found between layer lines in terms of POD, similarly to the current study. These differences may be explained by the overall higher POD in hens housed in cages which eases the detection of a possible difference between the layer lines. However, statistical analysis for the risk or presence of deviations was performed differently between the studies which may also explain different findings.

Taken together, our results confirm that KBD, mainly keel bone fractures, have a genetic component. Thus, it seems a promising approach to decrease the prevalence of keel bone fractures by selecting hens for a high bone stability as has been suggested by others (13, 67). In contrast, further selection on laying performance may amplify this animal welfare problem.

### Age and Keel Bone Damage

As hypothesized, KBD increased with age. This is consistent with other studies (5, 14, 16–19). However, several authors found that the prevalence of keel bone fractures peaked at about 50 weeks of age. Petrik et al. (4) assessed the prevalence of keel bone fractures on various farms in Ontario, Canada, with palpation. Fracture prevalence increased between 20, 35, and 50 weeks of age, but showed similar values at 50 and 65 weeks of age. Similarly, in an experimental study by Stratmann et al. (15), fracture prevalence, as assessed by palpation, increased with age but not beyond 52 weeks of age. Furthermore, Toscano et al. (21) found that the likelihood of experimental keel bone fractures increased with age but then began leveling off and to reverse at ~49.5 weeks of age. This was neither the case in the present study where prevalence increased until the end of the study nor in a previous study where we found more laying hens with keel bone fractures in the 72nd compared to the 51st week of age (19). However, in the present study, increase of prevalence of fractures and deviations was less pronounced at a higher compared to a younger age of the laying hens. The increase of prevalence was steepest between the 20th and 27th as well as between the 27th and 33rd week of age for deviations and between the 27th and 33rd as well as between the 33rd and 40th week of age for fractures, respectively. Thereafter, the increase was much less pronounced. Thus, it is possible that the keel bone is more susceptible to keel bone deviations and fractures until a certain age of about 40–50 weeks compared to higher ages. Further studies are required to get a better insight into the effect of age on KBD, especially into the possible mechanisms that may make the keel bone less susceptible to fractures and deviations after a certain age.

### Locomotor Activity

As hypothesized, locomotor activity differed between treatment groups over time. Locomotor activity decreased in non-egg laying hens (groups S and SE) toward the end of the study but not in egg laying hens (groups C and E) and, thus, reached higher levels in egg laying compared to non-egg laying hens. The increased general locomotor activity in egg laying compared to non-egg laying hens may be a result of nesting behavior in hens of groups C and E but not in hens of groups S and SE. Increased locomotor activity has been found prior to oviposition (44) and restlessness has been described as part of nesting behavior in laying hens (43). Interestingly, treatment with estradiol-17ß did not seem to have any influence on locomotor activity. Locomotor activity decreased in hens of group SE as it decreased in hens of group S and did not differ between these groups. Similarly, locomotor activity did not differ between groups C and E. Wood-Gush and Gilbert (42) found that nesting behavior could be induced by administration of estrogen and progesterone in ovariectomized hens while administration of estradiol alone only led to nesting behavior in a small part of the hens. Concerning intact laying hens, it has been found that exogenous estrogen did not have any influence on nesting behavior (68). Thus, the fact that no differences in locomotor activity were found between groups S and SE as well as between C and E could strengthen the assumption that the increased general locomotor activity in egg laying compared to non-egg laying hens was caused by the increase in activity related to nesting behavior. However, nesting behavior was not explicitly assessed in the present study and, thus, it cannot be assured whether the increased locomotor activity in egg laying compared to non-egg laying hens was related to nesting behavior.

Findings on locomotor activity of the two layer lines differed from findings concerning treatment groups. The low performing layer line G11 was more active at the beginning of the study while there were no differences between the layer lines once hens had started to lay eggs. As G11 showed a lower keel bone fracture prevalence compared to WLA it is possible that the increased activity in chicks and pullets in this layer line led to a higher breaking strength.

The method to compare locomotor activity between groups presented in this work is only a very first approach to get an idea about the general locomotor activity of the hens which may also be influenced by other factors. For example, the preferred location of each hen may influence the measurement as the antennae could not cover the whole pen so that hens that spent more time in the center of the pen are likely to have been registered more often compared to hens that spent more time at the boundary. Furthermore, no information about the use of different structures such as nest boxes and perches was acquired with this method.

It is possible that the differences in locomotor activity had an influence on keel bone health. Increased mobility positively influences bone strength (35, 36) but can also lead to an increased fracture prevalence due to a higher risk of collisions (7, 38). Furthermore, if nesting behavior was the cause for the increased locomotor activity in egg laying compared to non-egg laying hens, this may also have had a direct effect on keel bone damage as the possible competition for nest box access may lead to a higher fracture risk. Thus, the difference in locomotor activity may be a confounding factor when comparing keel bone fracture prevalence between egg laying and non-egg laying hens. However, as differences in fracture prevalence were extremely large between treatment groups while differences in locomotor activity were relatively small, we assume that a higher susceptibility to fractures of the keel bone due to egg production played a more important role in the etiology of keel bone fractures than higher risk of collisions due to increased activity in egg laying hens. Nevertheless, a more detailed assessment of the hens' activity and behavior is required in order to determine the relevance of the findings on locomotor activity in relation to keel bone damage. This could be done by video monitoring and should include information about the behavior performed by hens while moving, about the use of structures such as perches and nest boxes, and about the kind of movement they are performing. It should also include wing movements such as wing-flapping and balancing movements when perching or ascending and descending perches as these activities may have a larger effect on the keel bone than general locomotor activity which is likely to have most impact on leg bones.

### Limitations of This Study

Being the very first study to compare keel bone damage between hens in which egg production was suppressed and intact laying hens and, thus, including a number of methods that have not been applied in many studies before, the present study has some limitations that need to be kept in mind when drawing conclusions.

Sample sizes differed between groups. This was due to the very high costs of the estradiol-17ß implants which made it impossible to treat a higher number of hens with this implant. However, we decided not to orientate sample sizes of the other groups to the estradiol groups in order to have more hens to compare in groups S and C as our main focus was on differences between hens treated with deslorelin acetate and control hens. The differences in sample size also increased throughout the study due to higher mortality in groups E and SE compared to the other groups between sampling periods 6 and 7. Pathological investigations could not clarify whether mortality was associated with estradiol-17ß supplementation. Although an unbalanced sample size is not ideal, the methods used for statistical analyses do account for unbalanced sample sizes and allow to compare these groups and, thus, the influence of this limitation was kept as low as possible.

Sample size was also quite low in some groups, especially for analysis of keel bone fractures where only hens in which all radiographs could be assessed for fractures were included. Some radiographs could not be evaluated for fractures due to the legs overlying the keel bone when taking the radiograph. This occurred more often in non-egg laying compared to egg laying hens. Thus, sample size was lower in group S compared to group C for analysis of keel bone fractures. In future studies, radiographs could be taken with hens hanging upside down as shown by Sirovnik and Toscano (69) as well as Rufener et al. (70), which is likely to reduce the number of non-evaluable radiographs.

Furthermore, taking only lateral radiographs may have led to specific deviations, although being visible, being underestimated. However, postero-anterior radiographs of the keel bone, which would allow for a more detailed analysis of some kinds of deviations, are not useful as too many other parts of the body such as the vertebral column, being situated between the keel bone and the detector, overlie the keel bone and make the assessment of the keel bone impossible. Taken together, the assessment of the severity of deviations, i.e., POD, can only be an estimation of the actual amount of deviated keel bone area and must be interpreted with care, also because POD itself and differences in POD between the groups were small in the present study.

Control hens did not receive any sham operation or any placebo implant. Thus, it cannot fully be excluded that differences between treated and control hens were based on the treatment process itself rather than on egg production or estradiol-17ß plasma concentrations. However, although not being objectively quantified, treated hens did not show any evident behavioral changes after the implantation procedure. They fed and used the perches directly after having been brought back to the pen. Furthermore, all sampling procedures occurred throughout a broad time period while any possible effects of the small surgical procedure would only be expected to be relevant for a certain time after the procedure. In addition, hens of groups E, S, and SE were all subject to the same surgical procedure but hens of group E showed a higher prevalence of keel bone fractures compared to hens of groups S and SE. Thus, it is clear that differences between these groups are not caused by any effect of the surgical procedure and indicate that the procedure did not have a major influence on keel bone damage.

## Conclusions

The present study clearly shows a strong association between egg production and keel bone fractures. In order to find solutions to decrease fracture prevalence, possible changes in the bone caused by egg production should be analyzed. One characteristic that differed between egg laying and non-egg laying hens was the radiographic density. This may reflect a lack of bone mass in egg laying hens. However, further studies are required to strengthen this assumption. Furthermore, genetic background and, possibly, selection for high laying performance have been found to influence keel bone fractures, indicating that selection for high bone quality may be a promising way to decrease their prevalence. In addition, the early onset of lay may negatively influence keel bone health and it seems worth to have a closer look at this possible relationship as this trait could be easily manipulated in commercial farms.

## Data Availability Statement

The raw data supporting the conclusions of this article will be made available by the authors, without undue reservation, to any qualified researcher.

## Ethics Statement

The animal study was reviewed and approved by Lower Saxony State Office for Consumer Protection and Food Safety, Postfach 39 49, 26029 Oldenburg.

## Author Contributions

BE, LS, and SP conceived and designed the experiments. SW bred the chicks of the layer line G11. BE and SP performed the experiments. AP and BE analyzed the data. BE wrote the original draft of the manuscript and visualized the data. AP, SP, LS, CT-R, and SW reviewed and edited the original draft of the manuscript. All authors read and approved of the final manuscript.

### Conflict of Interest

The authors declare that the research was conducted in the absence of any commercial or financial relationships that could be construed as a potential conflict of interest.
